# Pharmacist intervention and identification of adverse events related to treatment efficacy in cancer chemotherapy to improve clinical outcomes

**DOI:** 10.1186/s40780-024-00403-4

**Published:** 2024-12-18

**Authors:** Hironori Fujii

**Affiliations:** Department of Pharmacy, Gifu University Hospital, 1-1 Yanagido, Gifu, 501-1194 Japan

**Keywords:** Cancer chemotherapy, Adverse events, Treatment efficacy, Evidence practice gap, Chemotherapy-induced nausea and vomiting, Acneiform rash, Hypomagnesemia, Neutropenia, Cancer cachexia

## Abstract

Adverse events (AEs) induced by cancer chemotherapy reduce not only patient quality of life (QOL) but also the efficacy of treatment. Management of AEs can therefore improve both the efficacy and safety of cancer chemotherapy. This review describes the contribution of pharmacists to the management of adverse events aimed at improving the treatment efficacy of cancer chemotherapy. Efforts to improve the evidence-practice gap are a useful approach to countermeasures against AEs. Pharmacists can intervene in these efforts in the course of their daily practice. Here, we made undertook to improve the evidence-practice gap in prophylaxis pharmacotherapy for chemotherapy-induced nausea and vomiting (CINV) and anti-EGFR antibody-induced acneiform rash. After intervention by pharmacists, the rate of adherence to prophylaxis pharmacotherapy for these AEs was significantly improved, and the incidence of CINV and acneiform rash was significantly decreased. Notably, time to treatment failure (TTF) with anti-EGFR antibody therapy tended to be increased, and may have contributed to an improvement in therapeutic effect. Next, we examined adverse events associated with anti-cancer drugs related to the therapeutic effect of cancer chemotherapy. Incidence of hypomagnesemia in patients receiving anti-EGFR antibodies and neutropenia in patients receiving TAS-102 was significantly associated with the therapeutic effect of cancer chemotherapy. Moreover, we examined the impact of cancer cachexia, a cancer-associated AE, on the therapeutic effect of immune checkpoint inhibitors. In patients receiving nivolumab, the presence of cancer cachexia prior to treatment initiation was associated with shorter OS and TTF. In summary, pharmacist management of AEs was shown to improve treatment response. Further, AEs which are predictive of treatment response in cancer chemotherapy were identified. Management of these AEs is an important role for pharmacists aiming to improve patient QOL and treatment efficacy.

## Introduction

As the number of people diagnosed with cancer continues to grow worldwide together with the aging of the population, early detection and improvements in diagnosis and treatment have resulted in an increase in the number of patients receiving cancer chemotherapy [1, 2]. Cancer drug therapy has various purposes. For early-stage cancer and chemotherapy for leukemia, lymphoma and other cancers that are likely to be cured by chemotherapy, preoperative and postoperative chemotherapy is aimed at cure, and includes thorough antitumor therapy and active supportive care to help patients tolerate the treatment. On the other hand, for advanced or recurrent cancers, life-prolonging or palliative treatment is performed with an emphasis on quality of life (QOL). Even in advanced or recurrent cancer, however, conversion surgery leading to radical cure may be possible if anticancer drug therapy is successful [3]. Providing the maximum dose intensity that patients can tolerate increases the possibility of achieving a goal as radical cure.

An important factor that reduces the dose intensity of cancer drugs is adverse events (AEs). Severe AEs reduce patient QOL and lead to dose reductions or withdrawal of cancer chemotherapy, thereby reducing dose intensity. Supportive care therefore plays an important role in maintaining dose intensity by preventing, treating, and providing care for AEs.

The author is a pharmacist who is certified in oncology pharmacy by the Japanese Society of Pharmaceutical Health Care and Sciences. He has conducted research on the evaluation of treatment efficacy in collaboration with physicians [4–6] and QOL evaluation [7–9], and clinical research on special populations [10, 11], mainly in the field of cancer. In this context, he has focused on the creation of clinical outcomes that contribute to the efficacy of cancer chemotherapy.

This review outlines (1) efforts to improve the evidence practice gap and its clinical outcome evaluation, and (2) the identification of AEs related to the treatment efficacy of cancer chemotherapy. The goal is to establish AE management to improve the treatment efficacy of cancer chemotherapy.

## Efforts to improve the evidence-practice gap by pharmacists and clinical outcome evaluation

Evidence-based medicine is the deliberative, explicit and judicious use of current best scientific evidence in making decisions about the care of individual patients [12, 13]. However, there is increasing recognition of gaps between best scientific evidence and clinical practice in many fields of healthcare, called the “evidence-practice gap” [14].

Clinical pharmacists are involved daily in pharmacotherapy for inpatients and outpatients, and are likely to encounter evidence-practice gaps in their regular pharmaceutical work. Efforts to alleviate the symptom of AEs by pharmacists acting to identify and reduce the gap between best evidence and clinical practice in the treatment and prevention of AEs in cancer chemotherapy will therefore likely improve the maintenance of the dose intensity of cancer drugs.

This section describes our two previous studies on clinical outcomes and reduction in the evidence-practice gap. These focused on the prevention of chemotherapy-induced nausea and vomiting (CINV) and anti-epidermal growth factor receptor (EGFR) antibody-induced acneiform rash.

### Clinical evaluation of improvement in adherence to guidelines for antiemetic medication in moderate emetic risk chemotherapy [15]

CINV is a serious AE that decreases patient QOL and impairs patient adherence to medication [16]. Clinical practice guidelines for the use of antiemetic agents to prevent CINV due to chemotherapy agents based on their emetic risk have been provided by the American Society of Clinical Oncology (ASCO) [17], Multinational Association of Supportive Care in Cancer/European Society for Medical Oncology (MASCC/ESMO) [18], National Comprehensive Cancer Network (NCCN) [19], and Japanese Society of Clinical Oncology (JSCO) [20]. However, several researchers have reported that these guidelines are not always followed in clinical practice [21 – 24]. An observational study in Japan reported that the adherence rate was extremely low, ranging from 7.2 to 28.8% in the acute phase, and 1.1–9.7% in the delayed phase [21]. Against this, several reports have shown that the rates of antiemetic control in adherent patients are significantly higher than in non-adherent patients [22 – 24].

We therefore investigated adherence to the JSCO guidelines for antiemetic medication and the control of CINV in patients with metastatic colorectal cancer (mCRC) receiving the first course of chemotherapy with moderate emetic risk (MEC), including an oxaliplatin/5-fluorouracil/levofolinate combination regimen (modified FOLFOX6), irinotecan therapy, and irinotecan/5-fluorouracil/levofolinate combination regimen (FOLFIRI).

**Fig. 1:**
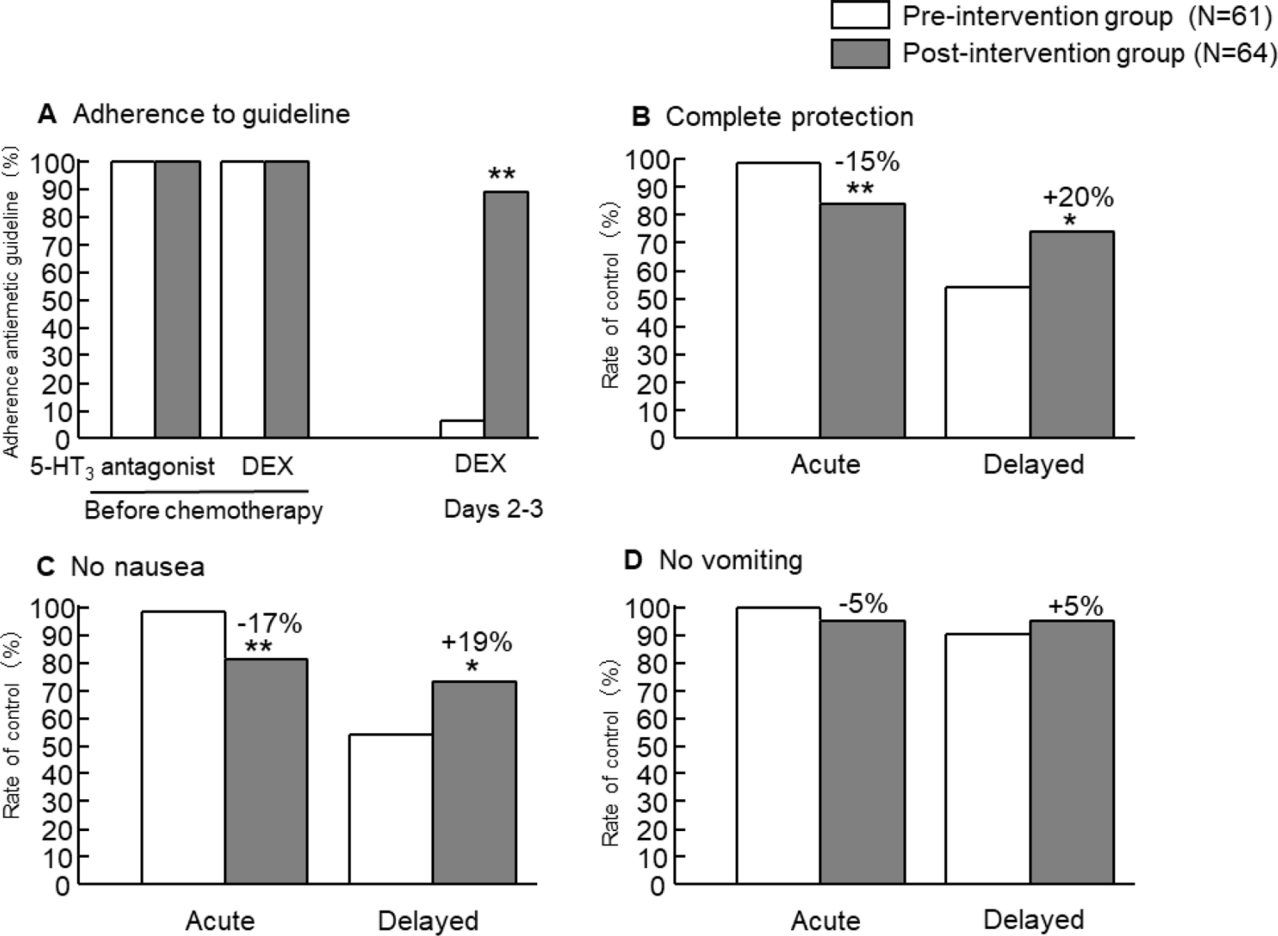
Comparison of adherence to the national guidelines for prevention of chemotherapy-induced nausea and vomiting (**A**), complete protection from nausea plus vomiting (**B**), nausea (**C**), and vomiting (**D**) in patients with colorectal cancer receiving moderately emetic chemotherapy (MEC) at an outpatient cancer chemotherapy clinic between the pre-intervention group and post-intervention group. Intervention was carried out to facilitate antiemetic medication based on clinical practice guidelines. *P < 0.05, ** *P* < 0.01 by Chi-square test

The rate of adherence to the antiemetic guidelines was 100% (61/61) in the acute period (within 24 h after chemotherapy), while that in the delayed period (2–5 days after chemotherapy) was only 6.6% (4/61) (Fig. 1A). Non-adherence was due mostly to the lack of dexamethasone (DEX) treatment on days 2 and 3. After an intervention by pharmacists in collaboration with physicians to increase the use of antiemetic medication based on the clinical practice guidelines, antiemetic medication adherence in the delayed period was markedly enhanced, to 89% (57/64), which led to significant enhancement in adherence (*P* < 0.05, Fig. 1B). However, the daily dose of DEX was 4 mg, lower than that recommended by the guidelines (8 mg), because CRC patients undergoing MEC typically receive prolonged chemotherapy every 2–3 weeks, which increases the risk of various side effects of DEX. Nevertheless, the rate of complete protection from nausea and vomiting during the delayed period was significantly increased, from 54 to 74% (*P* < 0.05).

For the acute phase, complete protection significantly decreased after the intervention, even though the guideline adherence rate was 100% both before and after the intervention. A possible reason for this may be that relative dose intensity (RDI) for MEC increased before and after the intervention (85.4% vs. 99.4%, *P* < 0.01). This is because the pharmacist intervention included not only appropriate use of antiemetic medication but also consultation with the physician following any decrease in the initial dose to ensure that the decrease was in fact necessary. Despite the increase in RDI, complete protection in the delayed phase increased. Of note, these findings may suggest that pharmacist intervention to improve the evidence practice gap can improve emetic control in patients with mCRC receiving MEC.

### Impact of minocycline and moisturizer prophylaxis for anti-EGFR antibody-induced acneiform rash on efficacy and safety [25]

Anti-EGFR antibodies induce characteristic AEs, including skin disorders, infusion reaction, and diarrhea. Among these, acneiform rash occurs most frequently from the early period of anti-EGFR therapy [26]. In severe cases (grade ≥ 3) of acneiform rash, treatment should be discontinued or the dose reduced [27]. Although the precise mechanisms underlying anti-EGFR antibody-associated acneiform rash are unclear, blockade of EGFR in cutaneous tissues may be involved, since EGFR is distributed throughout cutaneous tissues and is involved in the proliferation and differentiation of epidermal cells. In this regard, a positive correlation between cetuximab (Cmab) skin rash and tumor response rate and survival has been reported [28], and it is considered that the incidence and severity of skin rash is closely related to the efficacy of anti-EGFR antibody.

In a randomized phase II trial (STEPP trial) evaluating the efficacy of prophylactic skin therapy against Panitumumab (Pmab) skin toxicity in patients with mCRC, oral doxycycline (100 mg twice daily), a tetracycline antibiotic, combined with moisturizers, sunscreen, and a topical steroid (1% hydrocortisone cream) for 6 weeks prevented the development of grade 2 or higher skin toxicity, including pruritus, acneiform rash, and skin desquamation [29]. Based on this result, we collaborated with physicians to implement preventive measures using minocycline (100 mg/day) in combination with a moisturizer. We then compared the incidence of acneiform rash and the efficacy of cancer chemotherapy treatment between patients with prevention (prevention group) and those in whom measures were implemented after the acneiform rash appeared (non-prevention group).

**Fig. 2:**
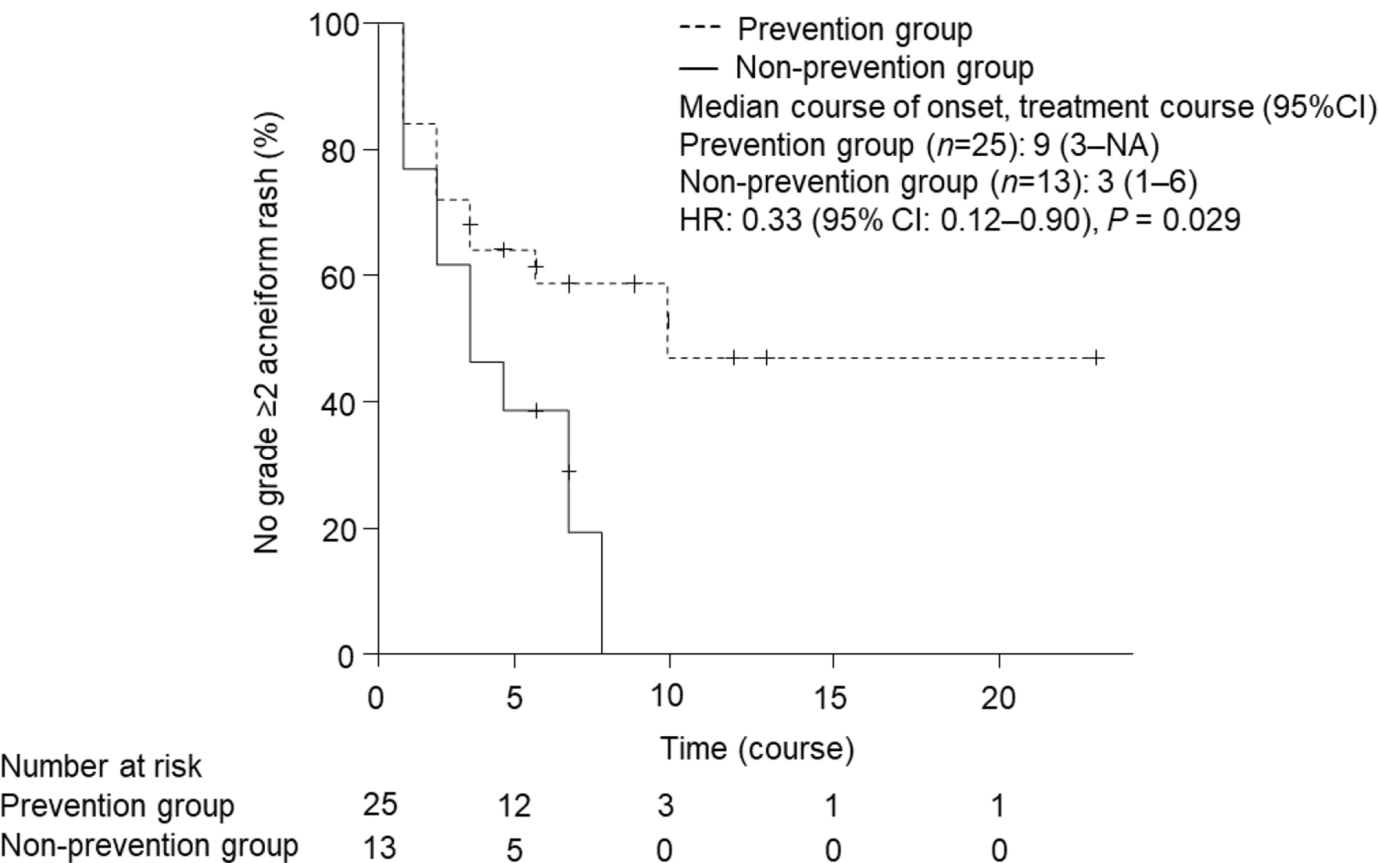
Comparison of time to development of grade ≥ 2 acneiform rash between the pre-emptive group and reactive group. Data were statistically compared by the log rank test

The incidence of grade ≥ 2 acneiform rash was significantly lower in the prevention group (44.0%) compared with the non-prevention group (84.6%) (*P* = 0.04). Time to development of grade ≥ 2 acneiform rash was significantly longer in the prevention group (*P* = 0.03, Fig. 2). Response rate (RR: complete response + partial response) tended to be higher in the prevention group (36.0% vs. 7.7%, *P* = 0.12) and time to treatment failure (TTF) tended to be longer in the prevention group (149.7 days vs. 110.2 days, *P* = 0.18).

This intervention to prevent acneiform rash with a minocycline-containing regimen was effective in reducing the incidence of acneiform rash induced by Pmab. The effectiveness of Pmab analyzed by TTF and RR tended to be better in the pre-emptive group than in the reactive group. Although the small number of cases in this study did not allow us to draw definite conclusions, the result suggests that suppressing acneiform rash may have a positive impact on treatment efficacy.

## Identification of adverse events associated with anti-cancer drugs which are related to the therapeutic effect of cancer chemotherapy

Some AEs associated with anticancer agents may involve the same mechanism as the pharmacological effect of the agent itself, and the occurrence of such AEs may correlate with treatment efficacy for cancer chemotherapy. If such AEs occur, high efficacy can be expected; on the other hand, if they become severe, they may lead to the interruption or discontinuation of treatment. The identification of an association between an AE and treatment efficacy suggests that countermeasures against the AE will lead to improved treatment efficacy. We conducted a retrospective observational study of hypomagnesemia caused by anti-EGFR antibodies, and neutropenia caused by Trifluridine-tipiracil (TAS-102) in relation to treatment response.

### Relationship between the incidence of hypomagnesemia caused by anti-EGFR antibodies and therapeutic effect [30]

Anti-EGFR antibodies such as Cmab and Pmab, when combined with FOLFOX or FOLFIRI as first-line chemotherapy, are effective in promoting survival in patients with mCRC; however, they often cause hypomagnesemia [31, 32]. Hypomagnesemia is associated with nausea and vomiting, fatigue, peripheral neuropathy, seizures, and QT prolongation, and severe cases lead to discontinuation of anti-EGFR therapy. Serum magnesium (Mg) concentrations are regulated by several transporters in the kidney, including transient receptor potential melastatin type 6 (TRPM6), which is stimulated by epidermal growth factor (EGF). Hypomagnesemia induced by anti-EGFR antibodies is therefore considered due to TRPM6 dysfunction [33].

In first-line chemotherapy for mCRC, prediction of treatment response is important for the continuation of treatment, because high tumor reduction may lead to conversion resection [3]. Therefore, we investigated the relationship between the incidence of hypomagnesemia and treatment response in patients with mCRC treated with anti-EGFR antibodies plus primary chemotherapy.

**Fig. 3:**
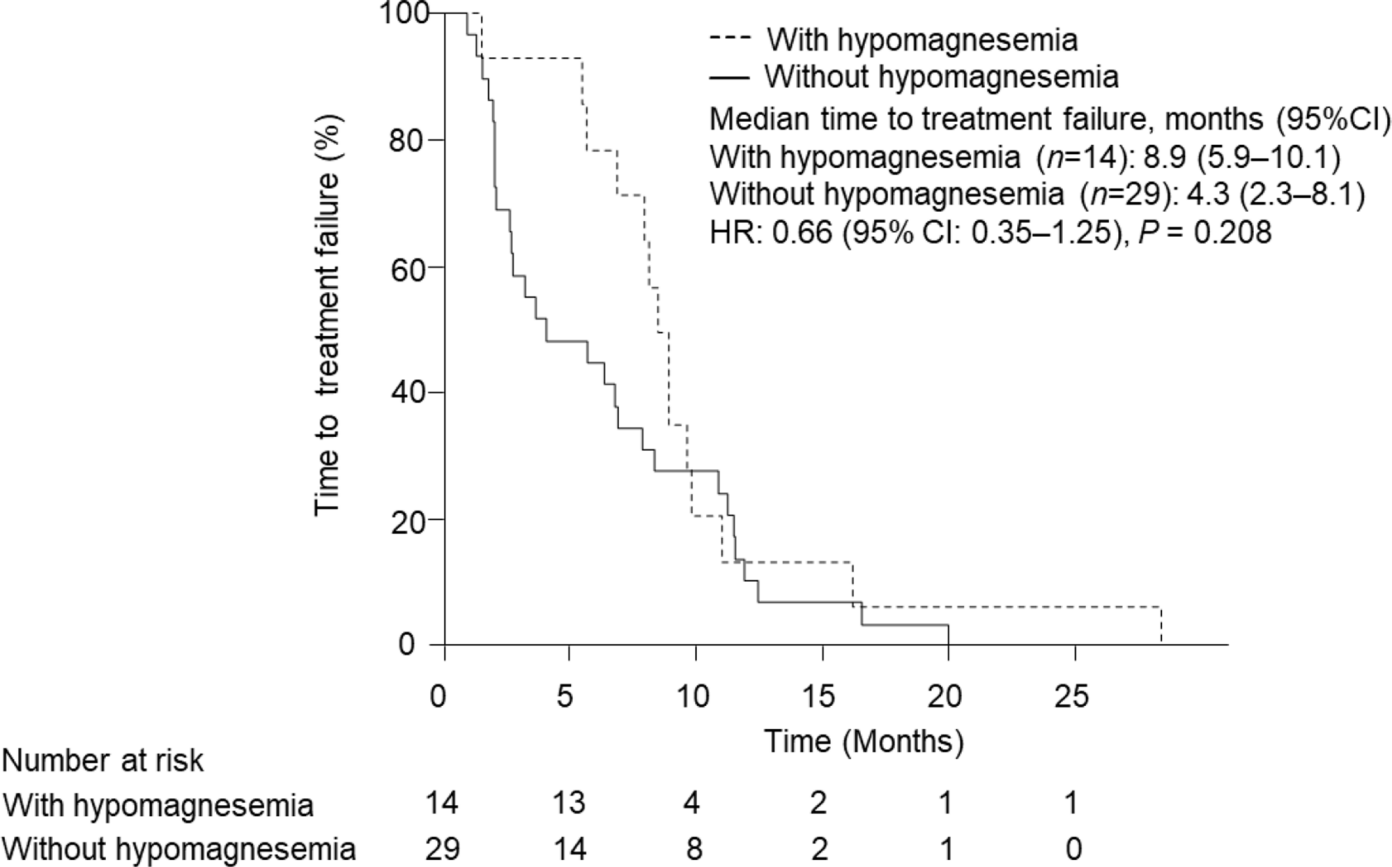
Kaplan–Meier plots comparing time to treatment failure (TTP) between patients with or without hypomagnesemia. Data were statistically compared by the log-rank test

Of 43 patients treated with anti-EGFR antibodies and primary chemotherapy, hypomagnesemia occurred in 14 patients (32.6%), consisting of 9 patients (20.9%) with grade 1 and 5 patients (11.6%) with grade 2. RR of tumor shrinkage was significantly higher in patients who developed hypomagnesemia (71.4% vs. 34.5%, *P* = 0.048); TTF also tended to be prolonged (Fig. 3: 273.5 days vs. 132 days, *P* = 0.208).

The relationship between the incidence of hypomagnesemia and efficacy of anti-EGFR antibodies is controversial; Vincenzi et al. [34] reported that the occurrence of Cmab-induced hypomagnesemia was closely associated with increased tumor response rate and prolonged survival. In contrast, Vickers et al. [35] showed that hypomagnesemia or a 20% or greater decrease in serum Mg concentration after initiation of Cmab therapy was associated with worse overall survival (OS). However, both studies included patients after second-line therapy. Worsening physical status due to severe hypomagnesemia in patients who have been refractory to multiple chemotherapy regimens may lead to decreased responsiveness to anti-EGFR antibodies. Taken together, these findings suggest that hypomagnesemia induced by anti-EGFR antibodies in combination with primary chemotherapy for mCRC is a predictive marker for treatment response.

### Association of TAS-102 and bevacizumab with severe neutropenia with immortality time bias [36]

In the RECOURSE study, TAS-102 was clinically superior to placebo in OS in patients with mCRC refractory to standard therapy, including fluoropyrimidine, irinotecan, and oxaliplatin [37]. The most common AE associated with TAS-102 was neutropenia, with 37.9% of patients who received TAS-102 experiencing grade ≥ 3 neutropenia [37]. Furthermore, the incidence of grade ≥ 3 neutropenia tended to be higher with the combination of bevacizumab (Bmab) and TAS-102 compared to TAS-102 monotherapy (67% vs. 38%) [38].

Several retrospective cohort studies have reported an association between the occurrence of neutropenia and long-term survival during TAS-102 monotherapy or Bmab + TAS-102 therapy [39, 40]. However, these findings should be interpreted with caution, as categorizing a group without neutropenia into a group with neutropenia after treatment initiation may lead to the problem known as immortality time bias [41].

We therefore adopted an approach which addressed immortality time bias (time-varying Cox regression model) to examine the association between the development of severe neutropenia and survival in mCRC patients treated with Bmab + TAS-102. OS was determined using Simon and Makuch’s modified Kaplan-Meier survival curve.

**Fig. 4:**
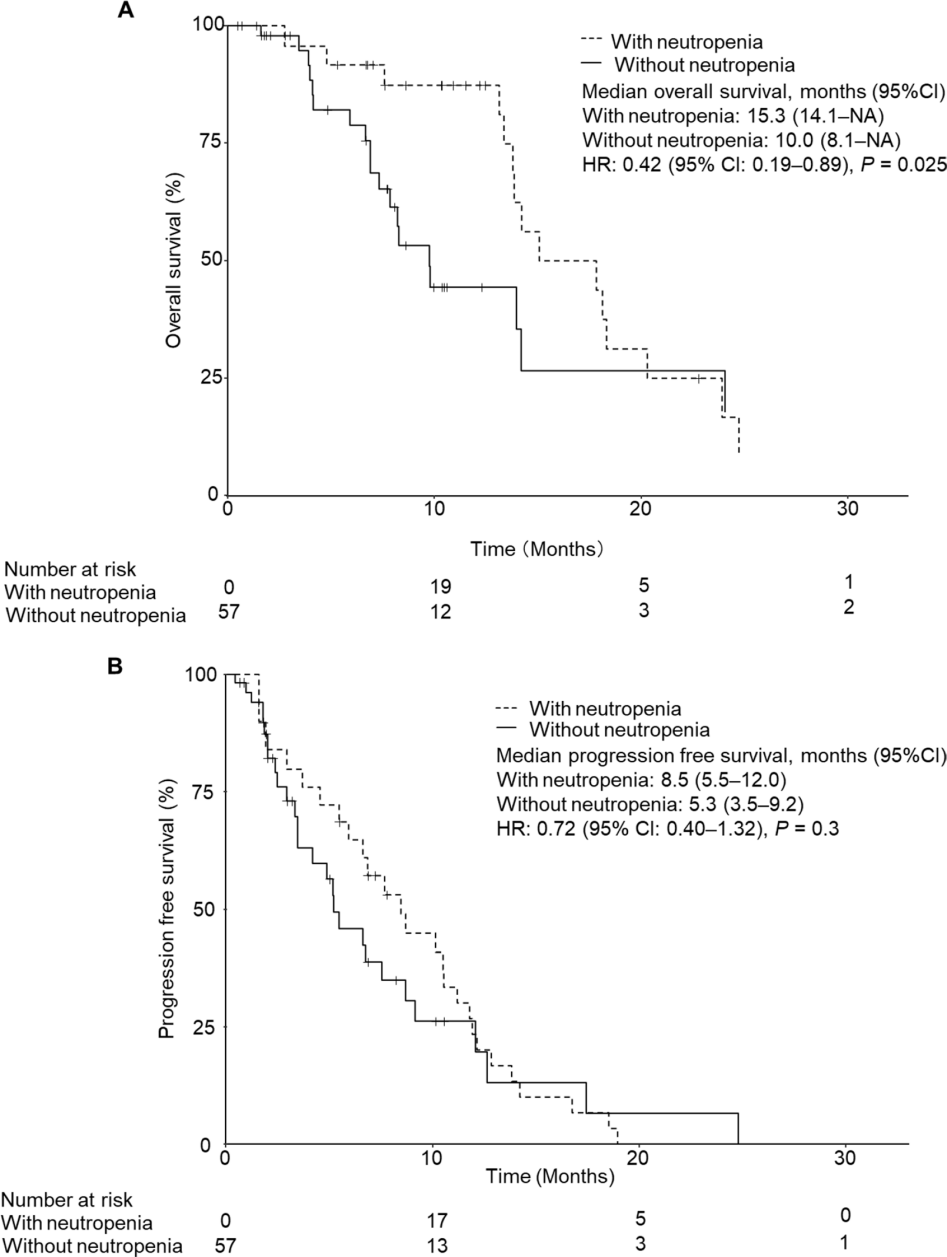
Simon and Makuch’s modified Kaplan–Meier curves for overall survival (**A**) and progression-free survival (**B**) in colorectal cancer patients who received a combination of trifluridine-tipiracil and bevacizumab. Data were statistically compared by time-varying Cox regression adjusting for age and modified Glasgow prognostic score (mGPS)

The results showed that OS was significantly prolonged in patients with severe neutropenia compared to those with non-severe neutropenia (15.3 months vs. 10.0 months, *P* < 0.05, Fig. 4A). Time-varying Cox regression showed that severe neutropenia was significantly associated with longer survival after adjustment for age and mGPS (HR: 0.42, *P* = 0.025). Median progression-free survival (PFS) was 8.5 months in patients with severe neutropenia and 5.3 months in those without (Fig. 4B). Although not significant, patients with severe neutropenia tended to have a longer PFS (HR: 0.72, *P* = 0.3).

In this study, patients with severe neutropenia had a significantly lower RDI for TAS-102 because the dose was reduced in response to severe neutropenia. However, disease control rates in these patients were higher. Although we as pharmacist are required to promote the appropriate dose reduction of anticancer drugs according to toxicity, these results suggest that dose adjustment based on dose reduction criteria in patients who develop severe neutropenia may be acceptable from a risk-benefit perspective, even if RDI is reduced.

TAS-102 has been shown to exert its anti-tumor immune effects primarily by directly eliminating tumor-associated macrophage 2 (TAM2) and promoting tumor growth through mechanisms such as angiogenesis and immunosuppression [42, 43]. Vascular endothelial growth factor (VEGF) inhibitors such as Bmab have been shown to enhance antitumor responses, by inhibiting infiltration of tumor-promoting immune cells, including TAM2. Neutrophils are also known to contribute to tumor growth by promoting angiogenesis and suppressing antitumor immune response [44, 45]. Accordingly, neutropenia induced by Bmab + TAS-102 may alter the tumor microenvironment and affect tumor progression after treatment.

## The impact of cancer-associated adverse events on the therapeutic effect of cancer chemotherapy [46]

Nivolumab, an immune checkpoint inhibitor (ICI), demonstrated a clinical benefit over placebo with respect to OS and PFS in advanced gastric cancer (AGC) after third-line treatment [47]. However, the RR in nivolumab treatment was only about 11.2%, and the disease control rate (DCR) was only 40%, with more than half of these patients failing to respond to treatment [47]. We therefore considered it necessary to predict which patients would have a satisfactory response to treatment with nivolumab.

In patients with non-small cell lung cancer (NSCLC), poor performance status (PS) [48] and decreased skeletal muscle mass [49] have been significantly associated with poor response to ICI. We hypothesized that the presence of cancer cachexia, which develops as a secondary disease in patients with cancer, underlies this poor PS and reduced skeletal muscle mass and is associated with the reduced efficacy of ICI. To evaluate cancer cachexia as a predictor of treatment response to immune checkpoint inhibitors, we examined the association between the presence of pretreatment cancer cachexia and treatment response in AGC patients treated with nivolumab.

**Fig. 5:**
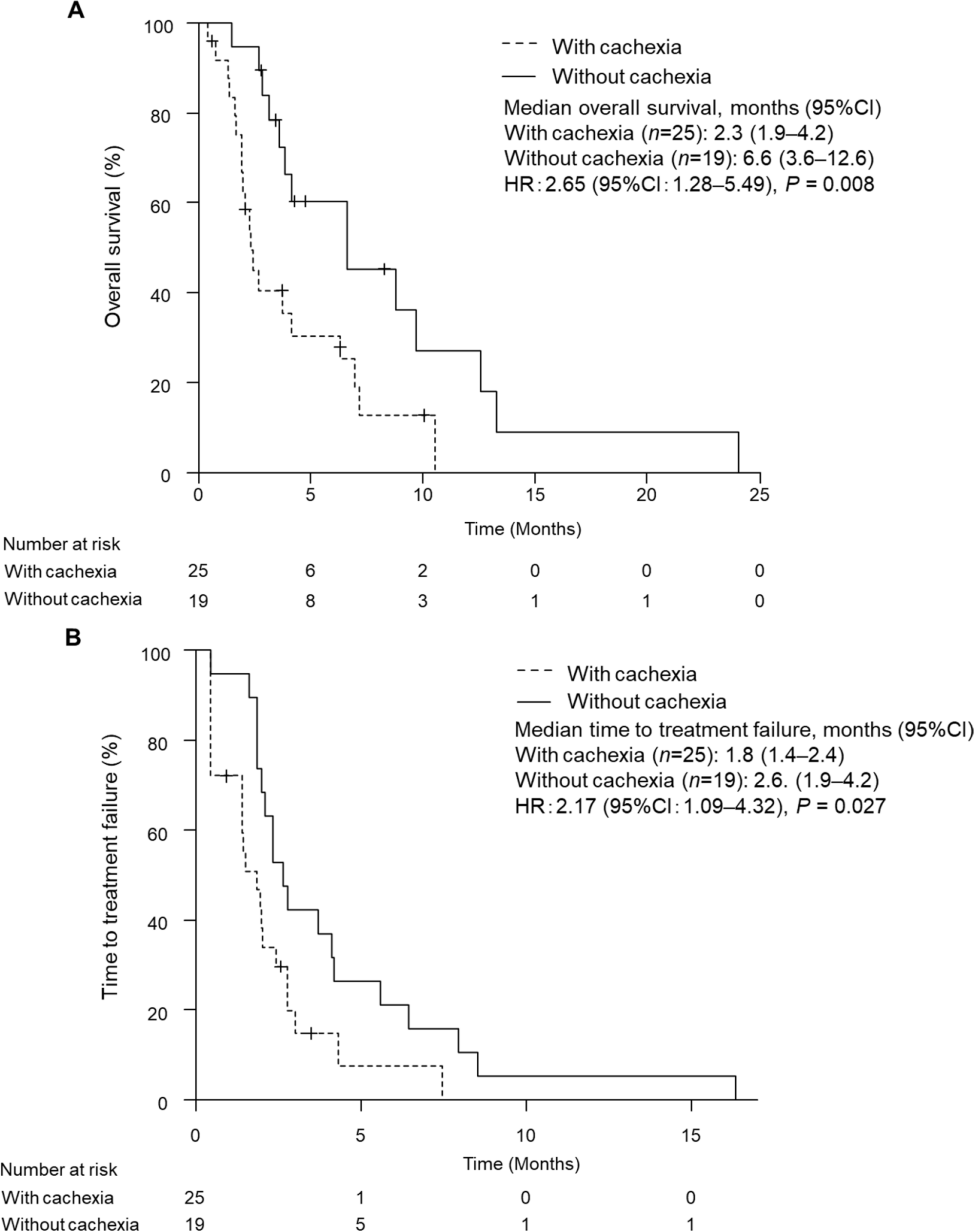
Kaplan-Meier curves for comparison of overall survival (**A**) and time to treatment failure (**B**) in advanced gastric cancer patients who received nivolumab with or without cancer cachexia. Data were statistically compared by the log-rank test

Forty-four AGC patients who received nivolumab were included in the study. After treatment with nivolumab, patients with cancer cachexia had significantly shorter OS and TTF (Fig. 5; OS, 2.3 months vs. 6.6 months, *P* = 0.008; TTF, 1.8 months vs. 2.6 months, *P* = 0.027). Cox proportional hazards regression showed a significant association between cancer cachexia and lower OS (HR: 2.34, *P* = 0.034) after adjustment for number of metastatic organs/sites (> 2), neutrophile-lymphocyte ratio (NLR) and modified Glasgow prognostic score (mGPS). One-year survival tended to be lower in patients with cancer cachexia (0% vs. 15.7%, *P* = 0.073).

**Fig. 6:**
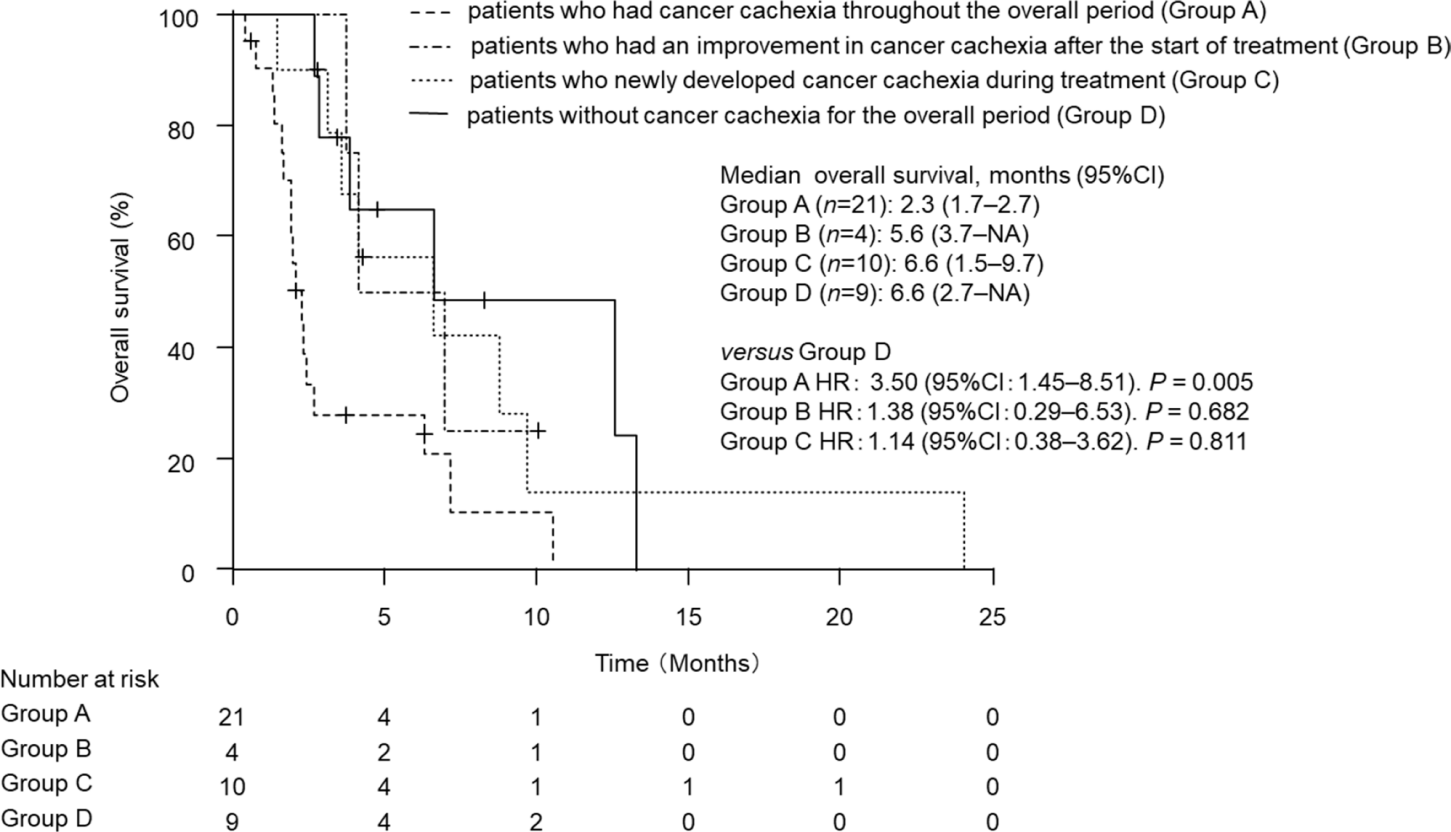
Kaplan–Meier curves for comparison of overall survival in advanced gastric cancer patients who received nivolumab among four groups: patients without cancer cachexia for the overall period (Group **A**), patients who newly developed cancer cachexia during treatment (Group **B**), patients who had an improvement in cancer cachexia after the start of treatment (Group **C**), and patients who had cancer cachexia throughout the overall period (Group **D**). Data were statistically compared by the log-rank test

The effect of the development or improvement of cancer cachexia after nivolumab initiation on OS was examined. Of the 25 AGC patients with cancer cachexia, 4 patients showed improvement in cachexia during nivolumab treatment. In contrast, in 19 patients without cachexia, 10 patients developed new cachexia during nivolumab treatment. Compared to patients without cancer cachexia throughout the entire period (group D: 9 patients), patients whose cancer cachexia improved after starting treatment (group B: 4 patients) and patients who developed new cancer cachexia during treatment (group C: 10 patients) did not show significant differences in OS (Fig. 6). However, patients who had cancer cachexia throughout the entire period (Group A: 21 patients) had significantly shorter OS compared to those in Group D (Fig. 6: 2.3 months vs. 6.6 months, *P* = 0.005).

Pressoir et al. reported that 63.2% of patients with upper digestive cancers, including esophageal, gastric, pancreatic, and liver cancer, had a weight loss of 10% or more at 6 months [50]. Furthermore, upper gastrointestinal cancer was independently associated with malnutrition in a multivariate analysis [50]. Cancer cachexia is a prognostic factor in gastric cancer patients [51], and in the present study was associated with shorter TTF as well as OS. It is possible that the immunostimulatory effect of nivolumab was not sufficient in AGC patients whose immunity was compromised by cancer cachexia.

As a pharmacological treatment for cancer cachexia, anamorelin, an orally active, high-affinity, selective ghrelin receptor agonist, has been shown to significantly increase lean body mass in patients with advanced NSCLC and gastrointestinal cancer, including gastric cancer [52]. Our results [46] and this finding [52] suggest that treatment of cancer cachexia using pharmacologic treatment with anamorelin may improve the efficacy of nivolumab in patients with AGC.

## Conclusion

In this review, we first addressed how pharmacist-led interventions aimed at closing evidence gaps reduced adverse events and improved treatment outcomes. Next, we focused on identifying adverse events that affected treatment efficacy and emphasized the critical role of pharmacists in managing these events to optimize therapeutic results. Finally, we examined cachexia as a condition that impaired therapeutic efficacy, rather than as a mere side effect. We highlighted the potential of interventions such as anamorelin to enhance outcomes in cachexia patients undergoing immune checkpoint inhibitor therapy. AE management in cancer patients by pharmacists is an important role that can not only improve patient QOL but also enhance treatment efficacy. We believe that these studies will contribute to further prolonging the lives of cancer patients and will make a significant contribution to the future development of medical pharmacy.

The content of this review is based on the routine work of pharmacists in clinical practice. Clinical questions must sometimes emerge in clinical practice, and pharmacists need to promote EBM, including meta-analysis of randomized controlled trial results. However, sufficient evidence can not always be obtained in real medical practice. Pharmacists need to develop the habit of constantly evaluating the results of clinical interventions such as prescribing suggestions in their daily pharmaceutical practice, which can provide useful evidence for patient care. The author hopes that pharmacists will widely conduct “research based on the routine work of pharmacists in clinical practice” in actual clinical settings in the future, as summarized in this review.

## Data Availability

Not applicable.

## Abbreviations

AEs: Adverse events
AGC: Advanced gastric cancer
Bmab: Bevacizumab
CINV: Chemotherapy-induced nausea and vomiting
Cmab: Cetuximab
DCR: Disease control rate
DEX: Dexamethasone
EBM: Evidence-based medicine
EGFR: Epidermal growth factor receptor
ICI: Immune checkpoint inhibitor
JSCO: Japanese Society of Clinical Oncology
mCRC: Metastatic colorectal cancer
MEC: Moderate emetic risk chemotherapy
mGPS: Modified Glasgow prognostic score
NLR: Neutrophile-lymphocyte ratio
NSCLC: Non-small cell lung cancer
OS: Overall survival
PFS: Progression-free survival
Pmab: Panitumumab
PS: Performance status
QOL: Quality of life
RDI: Relative dose intensity
RR: Response rate
TAM2: Tumor-associated macrophage 2
TAS-102: Trifluridine-tipiracil
TRPM6: Transient receptor potential melastatin type 6
TTF: Time to treatment failure
VEGF: Vascular endothelial growth factor
